# Lymph node dissection during cystectomy for non‐muscle‐invasive bladder cancer: A systematic review

**DOI:** 10.1002/bco2.70201

**Published:** 2026-04-28

**Authors:** Sean Lim, Lucielle Standish, Harrison Liu, Eldho Paul, Weranja Ranasinghe

**Affiliations:** ^1^ Department of Urology Monash Health Berwick Australia; ^2^ Department of Medicine, Nursing & Health Sciences Monash University Berwick Australia; ^3^ Monash Centre for Health Research and Implementation Monash University Berwick Australia

**Keywords:** bladder cancer, cystectomy, lymph node dissection, non‐muscle‐invasive bladder cancer, survival outcomes, systematic review

## Abstract

**Background and objective:**

Radical cystectomy (RC) is standard for muscle‐invasive disease (MIBC) and utilised frequently in high‐risk non‐muscle‐invasive bladder cancer (NMIBC). Pelvic lymph node dissection (PLND) is routinely performed during RC for MIBC, demonstrating a survival benefit. However, the oncologic value in NMIBC remains uncertain. As such, a systematic review was conducted to determine whether PLND confers an oncologic benefit in NMIBC patients undergoing RC.

**Materials and methods:**

A systematic search of MEDLINE, Embase and PubMed (January 1989–January 2025) was conducted in accordance with the Preferred Reporting Items for Systematic Reviews and Meta‐analyses guidelines (CRD42023443011). Eligible studies included NMIBC patients undergoing RC with or without PLND. Data extraction and quality assessment (ROBINS‐I, MINORS) were performed independently by two reviewers.

**Results:**

Twenty‐one retrospective studies (*n* = 35 793) met inclusion criteria. Lymph node positivity ranged from 0% to 13%. Comparative studies consistently demonstrated improved overall survival and cancer‐specific survival with PLND, particularly among pT1 subgroups (pooled 5‐year OS = 71.8%, 95% CI 59.3–84.3). Several studies demonstrated a dose–response association between lymph node yield or dissection extent and improved outcomes. Benefits were inconsistent for Ta/Tis disease. Pathological upstaging occurred in 16%–36% of clinically staged cohorts. However, study quality was moderate, with heterogeneity in PLND definitions, staging methods and adjuvant treatment use.

**Conclusions:**

PLND appears to improve staging and survival in high‐risk NMIBC, especially pT1 disease. Routine PLND for low‐risk Ta/Tis disease is unsupported. Standardised definitions of PLND extent and prospective evaluation are needed to confirm its therapeutic role.

## 1 INTRODUCTION

Radical cystectomy (RC), standard for muscle‐invasive bladder cancer (MIBC), is also considered in select patients with high‐risk non‐muscle‐invasive bladder cancer (NMIBC), particularly when intravesical therapy fails or variant histology is present.^1^, ^2^

Pelvic lymph node dissection (PLND) is routinely performed during RC in MIBC, demonstrating an overall 5‐year survival benefit of 6%–39%.^3^ While PLND is associated with complications such as lymphocele, DVT, vessel injury and nerve injury,^4^ its benefits, including improved staging accuracy and therapeutic effects, significantly outweigh the risks in MIBC.

While PLND is also routinely utilised with RC in NMIBC, the benefits of PLND to oncologic outcomes in this population remain uncertain.^5^

This systematic review aims to evaluate the oncologic and prognostic benefits of PLND in patients with NMIBC undergoing radical cystectomy. Specifically, it assesses oncologic outcomes such as cancer‐specific survival (CSS) and overall survival (OS) to inform clinical decision making in this subgroup.

## 2 METHODS

This systematic review is registered with the International Prospective Register of Systematic Reviews (PROSPERO ID: CRD42023443011) and was conducted in accordance with the Preferred Reporting Items for Systematic Reviews and Meta‐Analysis (PRISMA) Guidelines.

### 2.1 Search strategy

MEDLINE, Embase and PubMed databases were searched from 1989 until January 2025. Medical subject headings and keywords including ‘pelvic lymph node dissection’, ‘cystectomy’ and ‘non‐muscle invasive bladder cancer’ were combined with Boolean operators to identify all potentially relevant articles investigating the relationship between PLND and survival in NMIBC.

### 2.2 Study selection

Studies were included if they assessed survival outcomes of PLND during RC for NMIBC. NMIBC diagnosis was defined as being classified as stage Ta, Tis or T1 according to the American Joint Committee of Cancer (AJCC) classification, based on either clinical or pathological staging. All nodal dissection techniques were included. Outcomes of interest included OS and CSS. Studies were excluded if (1) the population only included MIBC, (2) the article did not address the listed primary survival outcomes, (3) NMIBC subgroup analysis was not done or unavailable, (4) it was a duplicate article, (5) it was a non‐English study and (6) only abstract with insufficient data was available. For studies with overlapping patient datasets (i.e. SEER, NCDB), only those with the largest dataset and longest recruitment period were included. Other duplicate articles were excluded unless overlap was minimal or their analyses offered distinct insights. Overlapping trials were omitted from meta‐analysis to prevent double counting.

Two reviewers (L.S., S.L.) independently screened titles and abstracts to identify relevant articles. The full texts of relevant articles were then evaluated against the strict inclusion and exclusion criteria. Disagreements between the reviewers were resolved by consensus.

### 2.3 Data extraction

Two independent reviewers (L.S., S.L.) extracted study details including population, interventions, lymph node involvement, follow‐up and adjuvant therapy. Objective outcomes were CSS and OS; 5‐year survival rates were extracted from Kaplan–Meier curves when not provided.

### 2.4 Quality assessment

Quality assessment was performed by two independent blinded authors (L.S., S.L.) using the Risk Of Bias In Non‐randomised Studies – of Interventions (ROBINS‐I) assessment tool for observational comparative trials and the Methodological Index For Non‐Randomised Studies (MINORS) checklist for single‐arm trials. The ROBINS‐I tool is used to assess the risk of bias across several domains, including confounding, patient selection, intervention, missing data and measurement of outcomes. The MINORS checklist provides a structured framework to appraise aspects including clarity of study aims, data collection methods, prospective design and adequacy of follow‐up, allowing consistent and comprehensive assessment of study quality. All conflicts were resolved by discussion.

### 2.5 Data synthesis

For narrative synthesis, studies would be grouped by outcome measurements (OS, CSS), NMIBC subgroups and staging method. To avoid double counting and sources of confounding, overlapping trials using registry data (i.e. NCDB, SEER) were not combined in the meta‐analysis. Similarly, pathological staging and clinical staging were analysed separately.

Inverse variance, random‐effects meta‐analyses were performed to assess pooled effects of interventions on individual outcomes. Random‐effects meta‐analyses using a DerSimonian and Laird estimator based on inverse variance weights were employed.^6^ Random‐effects meta‐analysis was chosen, as heterogeneity was anticipated due to variation in patient populations, tumour characteristics, method or extent of lymph node dissection or follow‐up duration. The presence or absence of heterogeneity was determined by the Cochran's *Q* test and the magnitude of heterogeneity was assessed by the *I* ^2^ statistic.

## 3 RESULTS

The electronic search yielded 2635 results (Figure S1). After removal of duplicates, title/abstract screening and full‐text screening, 23 studies were included for analysis. Articles were excluded for reasons such as the absence of NMIBC subgroup analysis (*n* = 34), abstract only with insufficient data (*n* = 20), incorrect population (*n* = 5), incorrect intervention/comparator groups (*n* = 2) and incorrect study design (*n* = 5). After data collection, a further one study was excluded due to overlapping patient populations from published databases.

### 3.1 Quality assessment

**FIGURE 1:**
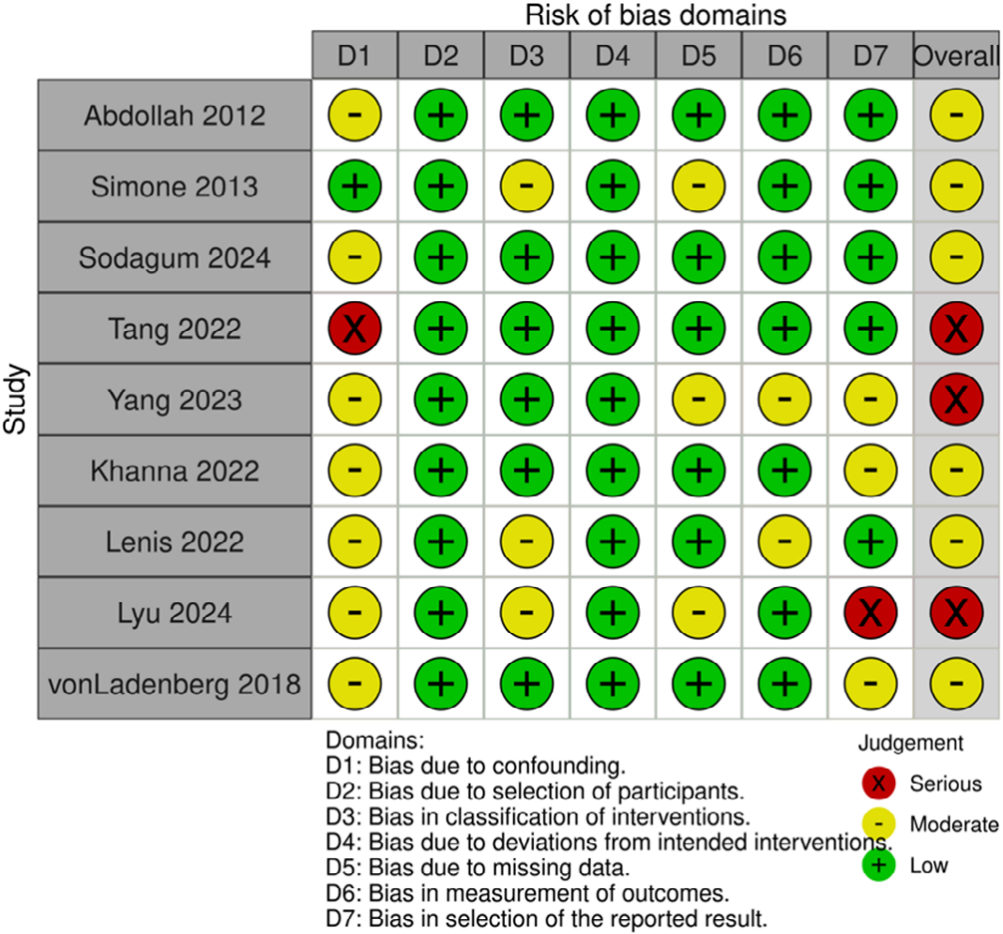
ROBINS‐I quality assessment for comparative studies.

**FIGURE 2:**
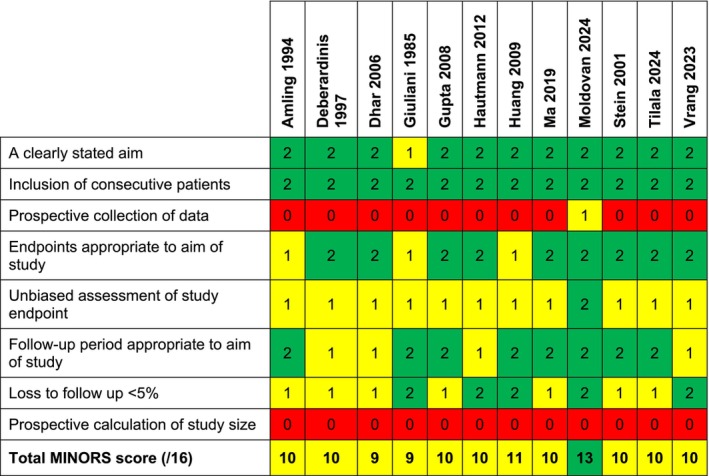
MINORS checklist quality assessment for non‐comparative studies.

Results from the quality assessment are summarised in Figures 1 and 2. Across comparative observational studies, risk of bias was assessed as moderate and serious for six and three studies, respectively. No included studies had an overall low risk of bias. Most studies had moderate (*n* = 7) or high (*n* = 2) confounding bias due to retrospective design and group differences. Across non‐comparative single‐arm trials, the quality was assessed as moderate (*n* = 11) or high (*n* = 1), with most studies losing points due to their retrospective design and the absence of pre‐study power calculations.

### 3.2 Study characteristics

The 21 studies conducted between 1985 and 2024 consisted of 35 793 patients (Table 1). All were retrospective cohort studies and investigated patients undergoing RC with PLND for NMIBC. Fourteen studies utilised pathological staging, and seven utilised clinical staging from TURBT specimens. Fifteen studies produced original data, whereas six investigated populations from known databases including SEER (*n* = 3) and NCDB (*n* = 3). Two studies with overlapping SEER data from 2004 to 2015 were included as one investigated both outcomes of OS and CSS,^21^, ^23^ and the other only investigated OS but provided subgroup analyses of all NMIBC cohorts (Ta, Tis, T1). Three studies with overlapping NCDB data were included. One study had the widest inclusion period (2014–2019) comparing survival outcomes in PLND compared with no PLND^19^; another study provided subgroup analyses of all NMIBC cohorts (Ta, Tis, T1), stratifying survival outcomes based on nodal yield^19^; and the third study performed a multivariate cox regression analysis based on nodal yield (<15 vs. >15 nodes),^25^ not performed in the prior two studies.

**TABLE 1:** Included characteristics.

Study (*z* = 21)	Study type	Population criteria	NMIBC subgroups	Intervention groups	Sample size (*n*)
Abdollah 2012^7^	Retrospective cohort	SEER Database (1988–2006). Non‐metastatic BC	pTa/Tis, pT1	PLND vs. no PLND ePLND (>10LN yield) vs. lPLND (<10LN yield)	11 183
Amling 1994^8^	Retrospective cohort	Primary NMIBC. Excluded MIBC and variant histology	pT0, pTa, pTis, pT1	All underwent PLND	220
DeBernadiis 1997^9^	Retrospective cohort	Primary bladder TCC	cT1	All underwent PLND	36
Dhar 2006^10^	Retrospective cohort	Non‐metastatic BC for curative treatment. Excluded variant histology, adjuvant therapy, positive margins	pT1	All underwent limited PLND	48
Giuliani 1985^11^	Retrospective cohort	Primary TCC of the bladder undergoing RC + bilat PLND	pT1	All underwent PLND	25
Gupta 2008^12^	Retrospective cohort	Curative treatment for ≥T2 disease OR NMIBC refractory to TURBT, intravesical CTx/ITx. Excluded variant histology, salvage cystectomy, inoperable disease	pT1	All underwent PLND	26
Hautmann 2012^13^	Retrospective cohort	Primary TCC of the bladder undergoing RC + bilat. PLND. Excluded Variant histology, neoadjuvant treatment, positive margins	pTa/Tis/T1, pTis, pTa, pT1	All underwent PLND	498
Huang 2009^14^	Retrospective cohort	Clinical stage CIS	cCIS	All underwent PLND	27
Khanna 2022^15^	Retrospective cohort	NMIBC. Excluded variant histology, neoadjuvant therapy, salvage/palliative cystectomy	cTa, cTis, cT1	Limited (<= 20 LNY) vs. extended (>20 LNY) PLND	1647
Lenis 2020^16^	Retrospective cohort	National Cancer Database (2004–2013): Non‐metastatic clinical stage Ta/is/1	cTa/Tis/T1, cTa, cTis, cT1	Lymph node yield <10 vs. > =10	3226
Lyu 2024^17^	Retrospective cohort	Radiologically node negative (<8 mm on CT/MRI) NMIBC	cTa/Tis/T1	No PLND vs. bilateral PLND	95
Ma 2019^18^	Retrospective cohort	Primary NMIBC. Excluded variant histology, lost to follow‐up	pT1	All underwent PLND	114
Moldovan 2024^19^	Retrospective cohort	National Cancer Database (2004–2019): Clinical stage NMIBC. Excluded preoperative nodal involvement or metastatic disease and prior BCG	cTa/Tis/T1, cTa, cTis, cT1	All underwent PLND	9399
Simone 2013^20^	Retrospective cohort	Non‐metastatic high‐grade UC. Excluded loss to follow‐up, neoadjuvant therapy	pT0/Tis/Ta/T1	Standard vs. extended PLND	234
Sodagum 2024^21^	Retrospective cohort	SEER Database (2004–2015). Non‐metastatic BC. Excluded variant histology	pTa/Tis/T1	Lymph node yield (1–10 vs. 11–20 vs. 21–30 vs. > 30)	2345
Stein 2001^22^	Retrospective cohort	Non‐metastatic BC. Excluded non‐bladder malignancy, salvage cystectomy	pT0/Tis/Ta, pT1	All underwent PLND	421
Tang 2022^23^	Retrospective cohort	SEER Database (2004–2015). Non‐metastatic Tis/Ta/T1 disease (urothelial or non‐urothelial origin)	pTa/Tis/T1, pTa, pTis, pT1	No PLND vs. limited (<10 LN yield) vs. extended (>/10 LN yield) PLND	1701
Tilala 2024^24^	Retrospective cohort	Primary TCC. Excluded variant histology and loss to follow‐up	pT1	All underwent PLND	18
von Landenberg 2018^25^	Retrospective cohort	National Cancer Database (2004–2012): Primary bladder TCC. Excluded unknown LNY, unknown chemotherapy status and nil follow‐up	cTa/Tis/T1	LNY < 10 vs. > =10 and <15 vs. > =15	4412
Vrang 2023^26^	Retrospective cohort	Non‐metastatic Primary TCC of the bladder undergoing robotic cystectomy + PLND for curative intent. Excluded salvage cystectomy	pT0/Ta/Tis/T1, pTa, pTis, pT1	All underwent extended PLND	130
Yang 2023^27^	Retrospective cohort (abstract)	Primary bladder TCC. Excluded adjuvant CTx	pTa/Tis/T1	No PLND vs. PLND	168

Indications for radical cystectomy included multifocal disease with significant tumour burden and high‐grade NMIBC refractory to TURBT, intravesical therapy, radiotherapy or chemotherapy. Seven studies excluded patients receiving neoadjuvant or adjuvant therapy. Sample sizes of NMIBC subgroups varied from 18 to 11 183. Outcomes measured included OS (*n* = 20) and CSS (*n* = 9). Specific tumour stage subgroups investigated included composite stages of Ta/Tis/T1 (*n* = 11) or Ta/Tis (*n* = 1), Ta (*n* = 7), Tis (*n* = 8) and T1 (*n* = 15).

Twelve studies included only patients undergoing PLND, four studies compared PLND to no PLND, four studies compared limited or standard PLND to extended PLND, and three studies compared subgroups of varying lymph node yields.^7^, ^8^, ^9^, ^10^, ^11^, ^12^, ^13^, ^15^, ^16^, ^17^, ^18^, ^19^, ^20^, ^21^, ^22^, ^23^, ^24^, ^25^, ^26^, ^27^, ^28^ Criteria for standard, limited and extended lymphadenectomy were based on various measurements including anatomical boundaries (*n* = 6) and number of lymph nodes retrieved (*n* = 9). For the remainder, descriptions of lymphadenectomy were not provided (*n* = 6).

### 3.3 Patient population

Median age ranged from 59.6 to 72.5 years. Lymph node involvement identified on histopathology varied from 0% to 13.1%.^7^, ^11^, ^29^ Seven studies did not provide details regarding nodal involvement.

Five studies did not provide information regarding whether neoadjuvant or adjuvant therapy was provided.^7^, ^19^, ^22^, ^23^, ^27^ Four excluded patients who underwent neoadjuvant or adjuvant therapy,^13^, ^15^, ^20^, ^30^ and one excluded patients where details regarding neoadjuvant treatment were not recorded.^25^ Eight studies had no patients undergoing neoadjuvant chemotherapy.^9^, ^11^, ^12^, ^14^, ^17^, ^21^, ^24^, ^26^ Four studies included patients receiving neoadjuvant or adjuvant therapy (radiotherapy, chemotherapy, mitomycin/thiotepa or BCG).^8^, ^16^, ^18^, ^25^ Details are summarised in Table 2.

**TABLE 2:** Population characteristics.

Study (*n* = 21)	Age (mean, range)	LN involvement in PLND groups (%)	Follow‐up duration	Neoadjuvant or adjuvant Tx
Abdollah 2012	72.5 (26–100)	**pTa/Tis + PLND:** Overall: 0.6% Limited: 0% Extended: 1% **pT1** **±** **PLND:** Overall: 5.2% Limited: 4.7% Extended: 5.8%	Not stated. KM curves demonstrated 10‐year follow‐up	Not provided
Amling 1994	63.0 (34–84)	5.9%	Overall median follow‐up 5 years (range 1 month to 18 years)	**Neoadjuvant RTx:** 74/220 **Mitomycin/thiotepa:** 46/220 **BCG:** 9/220
DeBerardinis 1997	**cT1:** 62.9 **cT1** **±** **Tis:** 63.3	cT1: 18.2% cT1 + Tis: 50%	Overall median follow‐up 120 months (range 15–180 months)	Nil
Dhar 2006	61.9 (31–84)	11.7%	Overall median follow‐up 45.1 (range 1.1–165.6) months	Nil (excluded)
Giuliani 1985	**<60:** 78 (39) **60–70:** 73 (36) **>70:** 51 (25)^a^	0%	FU data available for all patients until end of KM curve (10 years)	Nil
Gupta 2008	62.3 (30–85)	3.8%	Mean follow‐up of 66 months (8–120 months) and median follow‐up of 62 months	Nil
Hautmann 2012	64.3 (23–91)	Not provided for NMIBC subgroup	Median follow‐up of 38 months, mean follow‐up of 62 months (range 0–282 months)	Nil (excluded)
Huang 2009	64.2 (42–83)	3.7%	Median follow‐up of 94 months	Nil
Khanna 2022	68 (62–74)	6%	Median follow‐up of 4.1 years (IQR 2.3–7.8)	Nil (excluded)
Lenis 2020	65.3 (SD 11.0)	10.6	Median follow‐up of 39 months	**Neoadjuvant CTx:** 169/3226 (5.2)^a^ **Adjuvant CTx:** 324/3226 (10.0)^a^
Lyu 2024	66.5 (59–73)^b^	0%	Median follow‐up of 13.5 months	Nil
Ma 2019	64.8 (35–85)	9%	Median 133 months	**Adjuvant CTx:** 8
Moldovan 2024	< = 65 years: 3511 (37) >65 years: 5888 (63)^a^	**cTa:** 34/1019 (3.3)^a^ **cTis:** 26/507 (5.1)^a^ **cT1:** 832/6337 (13.1)^a^	**cTa:** 63.9 months (SD 42.6) **cTis:** 66.8 months (SD 45.6) **cT1:** 61.4 months (SD 44.0)	Not provided
Simone 2013	66.3 (SD 9)	Not provided for NMIBC subgroup	Not specified	Nil (excluded)
Stein 2001	66 (22–93)	pT0/Ta/Tis: 5% pT1: 14%	Overall: Median 10.2 years (Range 0–28)	Not provided
Sodagum 2024	69 (61–76)^b^	Not provided	Overall: Median 39 months (IQR 17–77)	Nil
Tang 2022	68 (60–74)^b^	4.0%	Overall: Median 77 months (IQR 42–116) PLND: 84 months (IQR 47–108) No PLND: 67 months (IQR 37–108)	Not provided
Tilala 2024	59.6 (45–85)	2/18 (11.1%)	Overall: Mean 56 (12–72) months, median 62 months	Nil
von Landenberg 2018	**LNY <10:** Mean age 69.26 (SD 10.32) **LNY > = 10:** Mean age of 67.02 (SD 10.48)	Not provided	Median follow‐up of 55.49 months (34.73–75.69)^b^	**Neoadjuvant CTx:** 200/4412 (4.5)^a^
Vrang 2023	68 (63–74)^b^	Not provided for NMIBC subgroup	Overall: Median follow‐up of 5.3 years (IQR 2.73–8.06)	Nil
Yang 2023	67.4 (SD 10.9)	3.4%	Median follow‐up of 24 months (13–43)	Not provided

^a^ Number (%).

^b^ Median +/− IQR.

### 3.4 Survival outcomes: Composite pathologic NMIBC

Six studies investigated OS for composite pathologic staged NMIBC subgroups (Table 3) (pTa/Tis/T1, Ta/Tis and pT0/Tis/T1).^7^, ^21^, ^22^, ^23^, ^26^, ^27^ Articles comparing the extent of PLND largely found higher OS in patients who underwent PLND. Five‐year OS ranged from 74.4% to 85% for PLND, compared with 62.3%–65% for non‐PLND cohorts. Three out of four articles performing multivariable Cox regression survival analysis found statistically significant improvements in OS for PLND groups versus non‐PLND cohorts. One study found higher mortality odds for no PLND groups (HR = 1.49, *p* < 0.05) compared with PLND cohorts for pTa or pTis disease.^7^

**TABLE 3:** Overall survival.

Overall survival	Stage subgroup	PLND overall survival (%)	No PLND overall survival (%)	Limited PLND overall survival (%)	Extended PLND overall survival (%)	Overall mortality vs. no PLND Cox regression MVA (HR)
Abdollah 2012	pTa/Tis	**5 years:** 74.4 (67.1–82.6) **10 years:** 53.4 (43.2–65.8)	**5 years:** 62.3 (54.7–70.9) **10 years:** 48.1 (39.8–58.1)	**5 years:** 78.2 (69.5–87.9) **10 years:** 63.6 (51.4–78.0)	**5 years:** 67.9 (55.3–83.3) **10 years:** 39.1 (24.9–61.3)	**No PLND (vs. PLND):** 1.49 (0.92–2.17), *p* < 0.05 **lPLND (vs. no PLND):** 0.5 (0.31–0.79), *p* < 0.05 **ePLND vs. no PLND:** 1.02 (0.64–1.62)
	pT1	5 years: 70.3 (66.6–74.1) 10 years: 57.7 (52.8–63.1)	5 years: 60.3 (55.4–65.6) 10 years: 41.4 (36.0–47.6)	5 years: 64.9 (59.9–70.3) 5 years: 51.2 (44.8–58.5)	5 years: 77.5 (72.5–82.8) 10 years: 66.7 (59.5–74.7)	**PLND (vs. no PLND):** 1.29 (1.06–1.57), *p* < 0.05 **lPLND (vs. no PLND):** 0.9 (0.73–1.12) **ePLND (vs. no PLND):** 0.61 (0.47–0.79), *p* < 0.001
Amling 1994	pTa	5 years: 88%^a^	—	—	—	—
	pTis	5 years:100%^a^	—	—	—	—
	pT0	5 years: 80%^a^	—	—	—	—
	pT1	5 years: 78%^a^	—	—	—	—
Dhar 2006	pT1	5 years: 83.1 (72.4–93.8)	—	—	—	—
Giuliani 1985	pT1	2 years: 80 5 years: 76 10 years: 63	—	—	—	—
Gupta 2008	pT1	5 years: 90	—	—	—	—
Hautmann 2012	pT1	5 years: 80^a^ 20 years: 34.3	—	—	—	—
Ma 2019	pT1	5 years: 60% 10 years: 49%	—	—	—	—
Stein 2001	pT0/T1/Tis	5 years: 84% +/− 3% (SE) 10 years: 67% +/− 4% (SE)	—	—	—	—
	pT1	5 years: 74% +/− 3% (SE) 10 years: 51% +/− 4% (SE)	—	—	—	—
Sodagum 2024	pTa/Tis/T1	**5 years** **LNY 1–10:** 68^a^ **LNY 11–20:** 76^a^ **LNY 21–30:** 86^a^ **LNY >30:** 81^a^	—	—	—	**LNY 11–20 (vs. LNY 1–10):** 0.81 (0.65–0.99), *p* < 0.043 **LNY 21–30 (vs. LNY 1–10):** 0.60 (0.45–0.79), *p* < 0.001 **LNY >30 (vs. LNY 1–10):** 0.66 (0.51–0.87), *p* = 0.004
Tang 2022	pTa/Tis/T1	5 years: 80^a^	5 years: 65^a^ Median 89 months	Median 111 months, *p* = 0.056	Median 151 months, *p* < 0.001	**lPLND (vs. no PLND):** 0.78 (0.65–0.93), *p* = 0.006 **ePLND (vs. no PLND):** 0.53 (0.45–0.62), *p* < 0.001
	pTa	5 years: 80^a^ Median 139 months, *p* = 0.148	5 years: 70^a^ Median 101 months, *p* = 0.148	Median 123 months, *p* = 0753 (vs. no PLND)	Median time not reached, *p* = 0.021 (vs. no PLND)	**PLND (vs. no PLND):** 0.74 (0.50–1.11)^b^
	pTis	5 years: 72^a^ Median 118 months, *p* = 0.617	5 years: 75^a^ Median 97 months, *p* = 0.617	Median 75 months, p = 0.415	Median 158 months, *p* = 0.201	**PLND (vs. no PLND):** 0.88 (0.54–1.44)^b^
	pT1	5 years: 79^a^ Median 139 months, *p* < 0.001	5 years: 62^a^ Median 82 months, *p* < 0.001	Median 115 months, *p* < 0.006	Median 150 months, *p* < 0.001	**PLND (vs. no PLND):** 0.60 (0.51–0.70)^b^
Tilala 2024	pT1	5 years: 87.58	—	—	—	—
Vrang 2023	pT0/Ta/Tis/T1	5 years: 85^a^	—	—	—	—
Yang 2023	pTa/Tis/T1	—	—	—	—	**PLND (vs. no PLND):** 1.32 (0.37–4.75), *p* = 0.667

^a^ Extracted from Kaplan–Meier curve.

^b^ Univariate log rank analysis, rather than multivariable Cox regression.

Tang 2022 found lower mortality in both limited (HR = 0.78, *p* = 0.006) and extensive PLND (HR = 0.53, *p* < 0.001) cohorts.^23^ Median survival was 89, 111 and 151 months for no PLND, lPLND and ePLND subgroups, respectively, which was statistically significant for ePLND on univariable analysis (*p* < 0.001).^23^ Sodagum et al., which used a similar SEER dataset to Tang et al., found that higher lymph node yields were associated with improved OS, with hazard ratios of 0.81 (*p* = 0.043), 0.60 (*p* < 0.001) and 0.66 (*p* = 0.004) for nodal yields of 11–20, 21–30 and >30, respectively, versus yields of 10 or less.^21^

Five studies investigated CSS for composite NMIBC subgroups (Table S1).^7^, ^13^, ^20^, ^21^, ^26^ Five‐year CSS ranged from 87.5% to 95% for PLND groups compared with 76.8% for no‐PLND in one study.^7^, ^13^, ^21^, ^26^, ^31^ One study reported no significant difference in CSS between sPLND and ePLND subgroups on univariate analysis.^20^ Two studies performing multivariate Cox regression analyses found statistically significant improvements in CSS for greater dissection.^7^, ^21^ One study found higher cancer‐specific mortality for the no PLND group (HR = 2.09, *p* < 0.05) and lower mortality for the limited PLND group (HR = 0.34, *p* < 0.05) but not for the extended PLND group (HR = 0.75, 95% CI 0.36–1.57).^7^ Another study found higher lymph node yields were associated with improved CSS, with hazard ratios of 0.92 (*p* = 0.636), 0.51 (*p* = 0.011) and 0.59 (*p* = 0.038) for nodal yields of 11–20, 21–30 and >30, respectively, when compared to yields of 10 or less.^21^

### 3.5 Survival outcomes: pTa

Two studies investigated OS for PLND in patients with pathological staged Ta disease (Table 3).^8^, ^23^ Five‐year OS rates were 80%^23^ and 88%,^8^ respectively. Tang et al. identified no statistically significant difference in OS for PLND versus no PLND cohorts. (HR = 0.74, CI 0.50–1.11).^23^ However, there was better OS for ePLND versus lPLND (*p* = 0.033) and no PLND (*p* = 0.021).^23^

Only one study investigated CSS for the Ta subgroup (Table S1), with a 5‐year CSS of 88%.^8^

### 3.6 Survival outcomes: pTis

Similar to Ta subgroups, two studies investigated OS for Tis (Table 3).^8^, ^23^ Five‐year OS rates were 72%^23^ and 100%,^8^ respectively. Once again, Tang et al. found no statistically significant differences in OS for PLND and no PLND cohorts (HR = 0.88, CI 0.54–1.44).^23^ There were also no survival differences for ePLND (*p* = 0.201) or lPLND (*p* = 0.415) compared with no PLND.^23^

### 3.7 Survival outcomes: pT1

Ten studies investigated OS for pT1 subgroups (Table 3).^7^, ^8^, ^10^, ^11^, ^12^, ^13^, ^18^, ^22^, ^23^, ^24^ Five‐year OS ranged from 60% to 90% in the setting of PLND. When no PLND was performed, OS ranged from 60.3% to 62%. Two studies comparing PLND to non‐PLND cohorts demonstrated statistically significant improved OS in PLND groups.^7^, ^23^ Tang et al. reported increased OS at 5 years for the PLND group of 79% versus the no PLND group of 62% (*p* < 0.001) and median survival of 139 versus 82 months, respectively.^23^ Abdollah et al. reported higher OS rates for the PLND group at 70.3% and 57.7% versus 60.3% and 41.1% at 5‐ and 10‐year post‐cystectomy, respectively.^7^ On multivariable analysis, both studies found PLND to be protective of OS, with one finding that omission of PLND resulted in higher overall mortality (HR = 1.29, CI 1.06–1.57, *p* < 0.05)^7^ and another finding lower overall mortality in PLND cohorts (HR = 0.60, CI 0.51–0.70, *p* < 0.05).^23^

Five‐year CSS rates ranged from 73% to 75.7% in three studies (Table S1).^7^, ^8^, ^18^ One study compared PLND subgroups and demonstrated higher CSS for PLND cohorts at 85.7% and 81.7% versus 77.5% and 70.0% for no PLND at 5 and 10 years, respectively.^7^ There were statistically significant reductions in overall mortality on multivariate Cox regression analysis for overall PLND (HR = 1.60, CI 1.18–2.17, *p* < 0.05) and ePLND groups (HR = 0.46, CI 0.30–0.70, *p* < 0.001), but once again not for lPLND (HR = 0.76, CI 0.54–1.06).^7^

### 3.8 Survival outcomes: Clinical staging

Seven studies utilised histopathological staging from TURBT/biopsy specimens (Table S2).^9^, ^14^, ^15^, ^16^, ^17^, ^19^, ^25^ One retrospective cohort (*n* = 1647) found pathological upstaging of pT2+ in 19.1% of patients.^15^ Another study utilising NCDB data (*n* = 3226) found pathological upstaging of pT2+ in 16.4% of NMIBC cystectomy cases.^16^

In the composite cTa/Tis/T1 group, one study reported lower OS in non‐PLND group versus PLND group (HR 0.68 [95% CI 0.62–0.74], *p* < 0.001).^19^ Another reported increased OS, CSS and DFS for NMIBC patients with LNY > 20 versus LNY < =20.^15^ A third study utilising NCDB data found nodal yields of 10 or more were associated with increased OS (HR = 0.85, 95% CI 0.76–0.95, *p* = 0.004) for patients not receiving neoadjuvant chemotherapy.^25^ This effect was more pronounced in the neoadjuvant chemotherapy subgroup (HR 0.49 [95% CI 0.28–0.83], *p* = 0.009).^25^ One study that investigated a similar NCDB dataset found significantly improved 5‐year OS for LNY > =10 versus LNY < 10 of 68.7% versus 60.6% (log rank *p* < 0.01).^16^

For the cTa subgroup, two trials identified no significant difference in OS between lPLND and ePLND.^15^, ^16^

For the cTis subgroup, 5‐year OS ranged from 60% to 83% between four studies.^14^, ^15^, ^16^, ^19^ A large registry analysis found no differences in OS between PLND and non‐PLND subgroups.^19^ Lenis compared LNY < 10 versus > =10 and did not find statistically significant differences (*p* = 0.12).^16^ Khanna found higher OS and CSS for LNY > 20 compared with LNY < =20, with 5‐year survival rates of 82% versus 60% and 94% versus 80% for OS and CSS, respectively.^15^

For the cT1 subgroup, 5‐year OS ranged from 60% to 70% between three trials.^15^, ^16^, ^19^ One study found significantly improved 5‐year OS in LNY > =10 versus LNY < 10 (69 vs. 60.0%, log rank *p* < 0.01).^16^ Another study (*n* = 1647) found higher 5‐year OS and CSS for LNY > 20 versus LNY < =20 (74 vs. 63% and 85 vs. 76%, respectively), but analyses identifying statistical significance were not performed.^15^

### 3.9 Meta‐analysis

**FIGURE 3:**
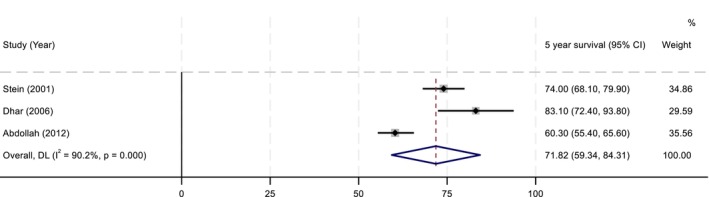
Meta‐analysis of 5‐year OS PLND pT1.

Due to significant statistical and methodological heterogeneity, as well as variable subgroups and outcomes assessed, the only 5‐year OS for PLND in pT1 disease was able to be combined. Five‐year OS was 71.82% (95% CI 59.34–84.31). *I* ^2^ was 90%, indicating significant heterogeneity (Figure 3).

## 4 DISCUSSION

Our findings support PLND as a consideration in certain patient groups with high‐risk NMIBC. Particularly, in pT1, high‐grade disease, there are potential therapeutic and staging benefits given observed high rates of pathological upstaging and nodal positivity. Benefits were less evident in pTa and pTis cohorts, consistent with their low metastatic risk. Although NMIBC traditionally has lower onco‐metastatic potential, high‐grade T1 and CIS may have higher risks of micro‐metastasis. Our findings align with emerging data that NMIBC, particularly aggressive T1 disease, may behave biologically like MIBC, with observed survival benefits with PLND.

PLND improves nodal staging accuracy, allowing appropriate adjuvant therapy allocation. The Will Rogers phenomenon suggests improved survival may reflect stage migration from better nodal identification and therapy selection, not direct therapeutic effect.^32^ While removing occult nodal metastases may directly improve oncological control, some of the observed survival advantage could be explained by improved identification of nodal involvement and appropriate upstaging and better selection of adjuvant therapy. Most studies in our review did not provide adjuvant therapy to patients (*n* = 17), either by practice or exclusion. In these trials, there were improved survival outcomes in PLND cohorts, potentially suggesting that PLND provides improved oncological control.

Despite variable PLND definitions, our results suggest a relationship between LNY and survival outcomes, with higher LNY associated with improved OS and CSS. These findings are incongruent with available evidence in MIBC. In two published randomised trials (LEA and SWOG), LND templates (i.e. limited vs. extended) were not associated with survival benefit.^33^, ^34^ Exploring this further, a recent study found higher LNs removed in limited LND arms of both of these trials compared to studies, potentially explaining the findings and supporting LN yield as a better marker than LND templates.^35^ Ta and Tis cohorts did not demonstrate statistically significant differences between limited and extended PLND,^7^, ^25^ likely explained by stage‐specific differences in nodal metastatic potential. In NMIBC, where nodal metastatic potential is stage dependent, the relative importance of nodal yield versus template remains unclear and warrants prospective evaluation.

Importantly, NMIBC is not a uniform disease, and there are multiple factors that confer higher risk in the absence of muscle invasion. For example, high‐grade T1 disease with lymphovascular invasion has been demonstrated to be biologically similar to MIBC.^36^, ^37^ Despite this, the presence of LVI was not consistently reported on across included studies. Including this factor may help to refine the indication for PLND with the broad NMIBC patient population. Additionally, BCG‐refractory Tis has higher likelihoods of occult nodal disease,^38^, ^39^ which may explain the survival benefit seen in included cohorts. Variant histologies (squamous, adenocarcinoma, sarcomatoid) carry higher metastatic risk.^40^, ^41^ Only a minority of included studies reported on variant histology, leaving unanswered whether survival benefits are driven by subsets of patients with aggressive biology.

PLND is associated with greater morbidity and perioperative risks, including increased operative time, higher blood loss, greater pain and immobility and higher risks of post‐operative complications such as lymphocele formation, DVT and more.^4^, ^42^ For lower‐risk disease (i.e. Ta/Tis disease), PLND provides more modest survival advantages. This must be weighed against the increased risks of extended pelvic surgery in the setting of lymphadenectomy. Unfortunately, none of the included studies investigated quality of life measures post‐operatively, which would be important in identifying morbidity differences and to make direct comparisons to survival benefit.

This review has several important limitations. All included studies were retrospective observational studies, prone to selection bias and confounding by indication; patients selected for PLND were often younger, fitter or treated in higher‐volume centres, which may have affected outcomes. A critical consideration when interpreting these findings is the potential for confounding by indication and selection bias inherent in retrospective cystectomy cohorts. In clinical practice, the decision to omit PLND is often deliberate. Patients who do not undergo PLND may be older, be frailer or undergo cystectomy for symptom control (e.g. refractory haematuria) rather than curative intent. These baseline characteristics are independently associated with worse OS and CSS. Consequently, poorer outcomes observed in the no PLND cohorts may reflect underlying patient risk rather than the oncologic impact of omitting PLND. Selection bias as a result of the retrospective nature of included studies is a possible explanation for the positive associated between LNY and survival, despite previously published research demonstrating the contrary.

Interpretation of the available evidence is limited by substantial heterogeneity in study design, patient populations, staging methods, PLND templates and adjuvant therapies, with the meta‐analysis demonstrating high heterogeneity (*I* ^2^ = 90%). Definitions of limited, standard and extended PLND varied widely, based on either anatomical boundaries or lymph node counts, and in several studies were not reported. The variability complicates direct comparison of oncological outcomes and limits the ability to determine whether survival differences are attributable to template extent, nodal yield or institutional practice patterns. Similarly, the use of neoadjuvant and adjuvant therapies was inconsistent or unreported in many cohorts. Because systemic therapy influences survival and may be guided by nodal staging, differences in adjuvant treatment allocation may confound the observed associations between PLND and oncological outcomes. Together, these sources of heterogeneity limit definitive conclusions regarding the independent therapeutic benefit of PLND in NMIBC.

Additionally, the power of our meta‐analysis was constrained by significant variability in the categorisation of nodal dissection, disease stage and reported outcomes (OS, CSS), resulting in only small numbers for statistical pooling within each subgroup. The overall quality of studies was variable, with risk of confounders including adjuvant therapy or variant histology. For clinical staged cohorts, high rates of pathological upstaging to ≥pT2, ranging from 19% to 36.6% demonstrated in some studies,^15^, ^16^may also serve as a significant confounder and hence another limitation of our study. Reporting of relevant prognostic factors—lymphovascular invasion, tumour grade and surgeon experience—was also inconsistent, further restricting meaningful adjustment. In addition, several studies drew from overlapping SEER or NCDB datasets, requiring authors of our review to exclude studies due to the possibility of data duplication and limiting the independence of findings. Finally, subgroup analyses were constrained by limited reporting, with few studies stratifying outcomes by variant histology, lymphovascular invasion or concomitant CIS, precluding more granular meta‐analysis and interpretation. In future, standardised, prospective, interventional trials are needed, comparing PLND subcategories, with consistent templates for extent of lymphadenectomy, and controlling for sources of bias.

## 5 CONCLUSION

This systematic review suggests that PLND during RC is associated with improved oncologic outcomes in patients with NMIBC, particularly those with pT1 or other high‐risk pathological features. The observed association appears to be both stage‐ and extent‐dependent, higher lymph node yields conferring greater benefit. However, the available evidence is derived exclusively from retrospective studies with significant heterogeneity in surgical templates and adjuvant treatment strategies, which limits causal inference regarding the therapeutic effect of PLND. In lower‐risk Ta and Tis disease, routine PLND appears to provide limited oncological value and must be weighed against increased operative complexity and morbidity. Overall, PLND should be considered a selective adjunct during radical cystectomy for high‐risk NMIBC rather than a universally beneficial intervention. Prospective, standardised studies are needed to clarify the independent therapeutic role of PLND, define optimal nodal yield thresholds and identify patient subgroups most likely to benefit.

## AUTHOR CONTRIBUTIONS

**Sean Lim:** Methodology; validation; formal analysis; investigation; data curation; writing—original draft; writing—review and editing; visualization; project administration. **Lucielle Standish:** Formal analysis; investigation; data curation; writing—original draft; writing—review and editing; visualization; project administration. **Harrison Liu:** Data curation. **Eldho Paul:** Formal analysis. **Weranja Ranasinghe:** Conceptualization; methodology; validation; writing—review and editing; supervision.

## CONFLICT OF INTEREST STATEMENT

All authors declare no conflicts of interest. This manuscript complies with the BJUI policy on declarations of interest. The corresponding author has collected and retained ICMJE Declaration of Interests forms from all co‐authors. According to these forms, none of the authors have any financial or non‐financial interests that could be perceived to influence the work. No authors have consultancy relationships, industry sponsorships, travel bursaries, meeting sponsorships or privileged access to new technologies relevant to this manuscript.

## Supporting information


**Figure S1:** PRISMA Flowchart
**Table S1:** Cancer Specific Survival
**Table S2:** Survival outcomes of clinical stage NMIBC

## References

[1] Holzbeierlein JM , Bixler BR , Buckley DI , Chang SS , Holmes R , James AC , et al. Diagnosis and treatment of non‐muscle invasive bladder cancer: AUA/SUO guideline: 2024 amendment. J Urol. 2024;211(4):533–538. 10.1097/JU.0000000000003846 38265030

[2] Daneshmand S . Determining the role of cystectomy for high‐grade T1 urothelial carcinoma. Urol Clin North Am. 2013;40(2):233–247. 10.1016/j.ucl.2013.01.003 23540781

[3] Bruins HM , Veskimae E , Hernandez V , Imamura M , Neuberger MM , Dahm P , et al. The impact of the extent of lymphadenectomy on oncologic outcomes in patients undergoing radical cystectomy for bladder cancer: a systematic review. Eur Urol. 2014;66(6):1065–1077. 10.1016/j.eururo.2014.05.031 25074764

[4] Loeb S , Partin AW , Schaeffer EM . Complications of pelvic lymphadenectomy: do the risks outweigh the benefits? Rev Urol. 2010;12:20–24. 20428290 PMC2859138

[5] van der Heijden AG , Bruins HM , Carrion A , Cathomas R , Compérat E , Dimitropoulos K , et al. European Association of Urology guidelines on muscle‐invasive and metastatic bladder cancer: summary of the 2025 guidelines. Eur Urol. 2025;87(5):582–600. 10.1016/j.eururo.2025.02.019 40118736

[6] DerSimonian R , Laird N . Meta‐analysis in clinical trials. Control Clin Trials. 1986;7(3):177–188. 10.1016/0197-2456(86)90046-2 3802833

[7] Abdollah F , Sun M , Schmitges J , Djahangirian O , Tian Z , Jeldres C , et al. Stage‐specific impact of pelvic lymph node dissection on survival in patients with non‐metastatic bladder cancer treated with radical cystectomy. BJU Int. 2012;109(8):1147–1154. 10.1111/j.1464-410X.2011.10482.x 21883849

[8] Amling CL , Thrasher JB , Frazier HA , Dodge RK , Robertson JE , Paulson DF . Radical cystectomy for stages Ta, Tis and T1 transitional cell carcinoma of the bladder. J Urol. 1994;151:31–35. 8254828 10.1016/s0022-5347(17)34865-6

[9] De Berardinis E , Giullianelli R , Seccareccia F , et al. Radical cystectomy for stage T1g3 transitional cell carcinoma of the bladder: long‐term follow‐up. Acta Urol. 1997;11:197–204.

[10] Dhar NB , Campbell SC , Zippe CD , Derweesh IH , Reuther AM , Fergany A , et al. Outcomes in patients with urothelial carcinoma of the bladder with limited pelvic lymph node dissection. BJU Int. 2006;98(6):1172–1175. 10.1111/j.1464-410X.2006.06502.x 16956353

[11] Giuliani L , Giberti C , Martorana G , Bonamini A , Natta GD , Rovida S . Results of radical cystectomy for primary bladder cancer. retrospective study of more than 200 cases. Urology. 1985;26(3):243–248. 10.1016/0090-4295(85)90119-0 4035840

[12] Gupta NP , Kolla SB , Seth A , Dogra PN , Hemal AK , Kumar R , et al. Radical cystectomy for bladder cancer: a single center experience. Indian J Urol. 2008;24(1):54–59. 10.4103/0970-1591.38604 19468360 PMC2684229

[13] Hautmann RE , de Petriconi RC , Pfeiffer C , Volkmer BG . Radical cystectomy for urothelial carcinoma of the bladder without neoadjuvant or adjuvant therapy: long‐term results in 1100 patients. Eur Urol. 2012;61(5):1039–1047. 10.1016/j.eururo.2012.02.028 22381169

[14] Huang GJ , Kim PH , Skinner DG , Stein JP . Outcomes of patients with clinical CIS‐only disease treated with radical cystectomy. World J Urol. 2009;27(1):21–25. 10.1007/s00345-008-0344-2 19066905

[15] Khanna A , Miest T , Sharma V , Campbell R , Hensley P , Thapa P , et al. Role of lymphadenectomy during radical cystectomy for nonmuscle‐invasive bladder cancer: results from a multi‐institutional experience. J Urol. 2022;207(3):551–558. 10.1097/JU.0000000000002266 34694143

[16] Lenis AT , Lec PM , Michel J , et al. Predictors of adequate lymph node dissection in patients with non‐muscle invasive bladder cancer undergoing radical cystectomy and effect on survival. Urol Oncol. 2020;38:796.e7–796.e14. 10.1016/j.urolonc.2020.04.027 32446641

[17] Lyu Q , Yang X , Cao Q , Zhuang J . Unveiling the necessity: should (very) high‐risk NMIBC patients undergoing RC opt for pelvic lymph node dissection?—a prospective cohort study. J Urol. 2024;211.

[18] Ma B , Li Y , Han S , Jiang X , Zhao Y , Guo J , et al. Radical cystectomy for pT1 urothelial carcinoma of bladder not amenable to TURBT: long‐term results. Eur J Surg Oncol. 2019;45(10):1993–1999. 10.1016/j.ejso.2019.07.018 31327502

[19] Moldovan M , Nam P , Satpathy Y , Wang L , Bagrodia A , Salmasi A , et al. Oncological and survival outcomes of pelvic lymph node dissection in patients with nonmuscle invasive bladder cancer undergoing radical cystectomy using the National Cancer Database. Clin Genitourin Cancer. 2024;22(6):102197. 10.1016/j.clgc.2024.102197 39260096

[20] Simone G , Papalia R , Ferriero M , Guaglianone S , Castelli E , Collura D , et al. Stage‐specific impact of extended versus standard pelvic lymph node dissection in radical cystectomy. Int J Urol. 2013;20(4):390–397. 10.1111/j.1442-2042.2012.03148.x 22970939

[21] Sodagum L , Passarelli R , Pfail J , et al. Pelvic lymphadenectomy: evaluating nodal stage migration and Will Rogers effect in bladder cancer. Urol Oncol. 2024;42:21.e9–21.e20. 10.1016/j.urolonc.2023.09.009 PMC10842630 37953186

[22] Stein JP , Lieskovsky G , Cote R , et al. Radical cystectomy in the treatment of invasive bladder cancer: long‐term results in 1,054 patients. J Clin Oncol. 2001;19:666–675. 11157016 10.1200/JCO.2001.19.3.666

[23] Tang Y , Wu K , Li X . Contemporary use trends and effect on survival of pelvic lymph node dissection for non‐muscle‐invasive bladder cancer. Front Surg. 2022;9:961430. 10.3389/fsurg.2022.961430 36034399 PMC9403057

[24] Tilala YM , Panda S , Tripathi A , Sharma S , Paul AS , Choudhuri S , et al. Long term outcomes and impact on renal function following radical cystectomy. Urologia. 2024;91(3):505–511. 10.1177/03915603241249231 38726742

[25] von Landenberg N , Speed JM , Cole AP , Seisen T , Lipsitz SR , Gild P , et al. Impact of adequate pelvic lymph node dissection on overall survival after radical cystectomy: a stratified analysis by clinical stage and receipt of neoadjuvant chemotherapy. Urol Oncol. 2018;36(2):78.e13–78.e19. 10.1016/j.urolonc.2017.10.021 29169845

[26] Vrang ML , Ostergren PB , Fode MM , Vangedal M , Lam GW . Robot‐assisted radical cystectomy with intracorporeal urinary diversion: a Danish 11‐year series. BJU Int. 2023;132(4):428–434. 10.1111/bju.16098 37395155

[27] Yang X , Li K , Zhuang J , Cai J , Wu Q , Yuan B , et al. The prognostic effect of pelvic lymph node dissection on the patients undergoing radical cystectomy. Chin J Urol. 2023;44.

[28] Barnes CM , Huang S , Kaipainen A , et al. Evidence by molecular profiling for a placental origin of infantile hemangioma. Proc Natl Acad Sci USA. 2005;102:19097–19102. 10.1073/pnas.0509579102 16365311 PMC1323205

[29] Xia LCR , Taylor B , Guzzo T . Lymph node dissection and survival in patient with clinical T1 high grade bladdere cancer undergoing radical cystectomy. J Urol. 2018;199.

[30] Chawla PC , Chawla A , Chaudhary S . Knowledge, attitude & practice on human papillomavirus vaccination: a cross‐sectional study among healthcare providers. Indian J Med Res. 2016;144(5):741–749. 10.4103/ijmr.IJMR_1106_14 28361828 PMC5393086

[31] Carsetti A , Aya HD , Pierantozzi S , Bazurro S , Donati A , Rhodes A , et al. Ability and efficiency of an automatic analysis software to measure microvascular parameters. J Clin Monit Comput. 2017;31(4):669–676. 10.1007/s10877-016-9928-3 27586243

[32] Sormani MP . The Will Rogers phenomenon: the effect of different diagnostic criteria. J Neurol Sci. 2009;287(Suppl 1):S46–S49. 20106348 10.1016/S0022-510X(09)71300-0

[33] Gschwend JE , Heck MM , Lehmann J , Rübben H , Albers P , Wolff JM , et al. Extended versus limited lymph node dissection in bladder cancer patients undergoing radical cystectomy: survival results from a prospective, randomized trial. Eur Urol. 2019;75(4):604–611. 10.1016/j.eururo.2018.09.047 30337060

[34] Lerner SP , Tangen C , Svatek RS , et al. Standard or extended lymphadenectomy for muscle‐invasive bladder cancer. N Engl J Med. 2024;391:1206–1216. 39589370 10.1056/NEJMoa2401497 PMC11599768

[35] Al‐Nader M , Krafft U , Darr C , et al. Impact of extended versus limited lymph node dissection on surgical outcome, recurrence patterns and survival after radical cystectomy. Clin Genitourin Cancer. 2025;23(3):102337. 10.1016/j.clgc.2025.102337 40234124

[36] Werntz RP , Smith ZL , Packiam VT , Smith N , Steinberg GD . The impact of lymphovascular invasion on risk of upstaging and lymph node metastasis at the time of radical cystectomy. Eur Urol Focus. 2020;6:292–297. 30297221 10.1016/j.euf.2018.09.019

[37] Elzayat EA , Al‐Zahrani AA . Pelvic lymphadenectomy in the treatment of invasive bladder cancer: literature review. Ther Adv Urol. 2011;2011:701481. 10.1155/2011/701481 PMC3166563 21904544

[38] Chehroudi AC , Black PC . Emerging intravesical therapies for the management of bacillus Calmette‐Guerin (BCG)‐unresponsive non‐muscle‐invasive bladder cancer: charting a path forward. Can Urol Assoc J. 2020;14(6):204–213. 10.5489/cuaj.6101 31977307 PMC7654668

[39] von Rundstedt FC , Lerner SP . Bacille‐Calmette‐guerin non‐responders: how to manage. Transl Androl Urol. 2015;4:244–253. 26816828 10.3978/j.issn.2223-4683.2015.05.03 PMC4708234

[40] Klaile Y , Schlack K , Boegemann M , Steinestel J , Schrader AJ , Krabbe LM . Variant histology in bladder cancer: how it should change the management in non‐muscle invasive and muscle invasive disease? Transl Androl Urol. 2016;5(5):692–701. 10.21037/tau.2016.06.13 27785426 PMC5071184

[41] Chalasani V , Chin JL , Izawa JI . Histologic variants of urothelial bladder cancer and nonurothelial histology in bladder cancer. Can Urol Assoc J. 2009;3(6 Suppl 4):S193–S198. 10.5489/cuaj.1195 20019984 PMC2792446

[42] Cheng H , Clymer JW , Po‐Han Chen B , Sadeghirad B , Ferko NC , Cameron CG , et al. Prolonged operative duration is associated with complications: a systematic review and meta‐analysis. J Surg Res. 2018;229:134–144. 10.1016/j.jss.2018.03.022 29936980

