# Supplementary material for: Lymph node dissection during cystectomy for non‐muscle‐invasive bladder cancer: A systematic review
Source: BJUI Compass. 2026 Apr 28;7(5):e70201. doi: 10.1002/bco2.70201 (PMC13124444; doi:10.1002/bco2.70201)
Supplement: Supplementary file 1 — Figure S1: PRISMA Flowchart Table S1: Cancer Specific Survival Table S2: Survival outcomes of clinical stage NMIBC [file BCO2-7-e70201-s001.docx]

**Supplementary Material**


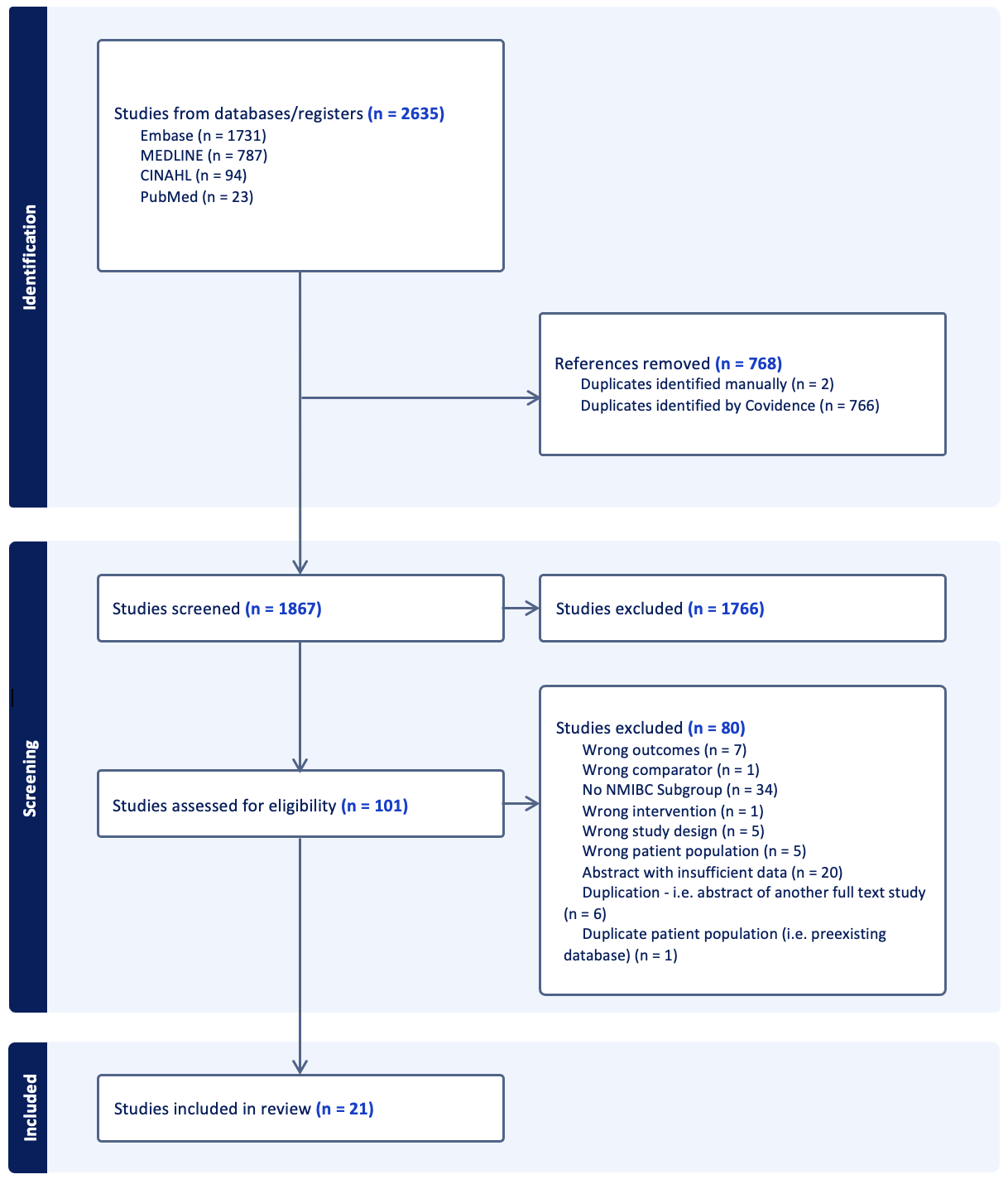


**Supplementary Figure 1:** PRISMA Flowchart

| **Cancer Specific Survival** | **Stage Subgroup** | **PLND Cancer Specific Survival (%)** | **No PLND Cancer Specific Survival (%)** | **Limited PLND Cancer Specific Survival (%)** | **Extended PLND Cancer Specific Survival (%)** | **Cancer Specific Mortality vs No PLND Cox Regression MVA (HR)** |
| --- | --- | --- | --- | --- | --- | --- |
| **Abdollah 2012** | pTa/Tis | 5 year: 88.9 (83.4-94.7)  10 year: 80.4 (71.2-90.8) | 5 year: 76.8 (69.9-84.5)  10 year: 71.9 (64.1-80.7) | 5 year: 90.2 (83.7-97.1)  10 year: 85.7 (75.6-97.0) | 5 year: 86.6 (76.7-97.7)  10 year: 70.8 (54.2-92.4) | **No PLND (vs PLND):** 2.09 (1.16–3.79), P<0.05  **lPLND (vs no PLND):** 0.34 (0.16–0.74)  **ePLND vs no PLND:** 0.75 (0.36–1.57) |
|  | pT1 | 5 year: 85.7 (82.8-88.7)  10 year: 81.7 (77.8-85.8) | 5 year: 77.5 (73.1-82.2)  10 year: 70.0 (64.7-75.7) | 5 year: 82.6 (78.3-87.0)  10 year: 78.7 (73.5-84.2) | 5 year: 89.6 (85.8-93.5)  10 year: 85.8 (80.3-91.7) | **No PLND (vs PLND):** 1.60 (1.18–2.17), P<0.05  **lPLND (vs no PLND):** 0.76 (0.54–1.06)  **ePLND vs no PLND:** 0.46 (0.30–0.70), P<0.001 |
| **Amling 1994** | pTa | 5 year: 88  10 year: 75 | - | - | - | - |
|  | pTis | **5 year:** 100 **10 year:** 92 | - | - | - | - |
|  | pT0 | **5 year:** 80 **10 year:** 66 | - | - | - | - |
|  | pT1 | **5 year:** 76 **10 year:** 62 | - | - | - | - |
| **Hautmann 2012** | pTis/Ta/T1 | **5 years:** 92.7% **10 years:** 90.5% **20 years:** 88.9% | - | - | - | - |
| **Ma 2019** | pT1 | 5 years: 73% 10 years: 70% | - | - | - | - |
| **Simone 2013** | pT0/Ta/Tis/ T1 | - | - | No statistically significant difference on KM between sPLND and ePLND. Log–rank P=0.229 | No statistically significant difference on KM between sPLND and ePLND. Log–rank P=0.229 | - |
| **Sodagum 2024** | pTa/Tis/T1 | **5 years**  **LNY 1-10:** 87.5**  **LNY 11-20:** 89**  **LNY 21-30:** 93**  **LNY >30:** 93** | - | - | - | **LNY 11-20 (vs LNY 1-10):** 0.92 (0.65−1.30), p=0.636  **LNY 21-30 (vs LNY 1-10):** 0.51 (0.31−0.86), p=0.011  **LNY >30 (vs LNY 1-10):** 0.59 (0.36−0.97), p =0.038 |
| **Vrang 2023** | pT0/Ta/Tis/ T1 | 5 years: 95** | - | - | - | - |

**Extracted from Kaplan Meier Curves

**Supplementary Table 1:** Cancer Specific Survival

| **Clinical Staging Studies** | **Clinical Stage Subgroup** | **PLND Overall Survival (%)** | **PLND Cancer Specific Survival (%)** |
| --- | --- | --- | --- |
| DeBerardinis 1997 | cT1/T1 + CIS | **5 years:** 73  **10 years:** 67, p<0.001  **Mean:** 8.9 years | 5 years 64**  10 years: 61.1 |
|  | cT1 | **10 years:** 80  **Mean:** 10.1 years | 5 years: 88** |
|  | cT1 + CIS | **10 years:** 46  **Mean:** 6.5 years | - |
| Huang 2009 | Tis | 5 years: 83  10 years: 56 | - |
| Khanna 2022 | Ta | **LNY <=20:**  5 years: 73  10 years: 46  **LNY >20:**  5 years: 72  10 years: 57 | **LNY <=20:**  84 = 5 years  70 = 10 years  **LNY>20:**  81 = 5 years  75 = 10 years |
|  | Tis | **LNY <=20:**  5 years: 60  10 years: 33  **LNY >20:**  5 years: 82  10 years: 66 | **LNY <=20:**  80 = 5 years  65 = 10 years  **LNY>20:**  94 = 5 years  87 = 10 years |
|  | T1 | **LNY <=20:**  5 years: 63  10 years: 45  **LNY >20:**  5 years: 74  10 years: 60 | **LNY <=20:**  76 = 5 years  70 = 10 years  **LNY>20:**  85 = 5 years  78 = 10 years |
| Lenis 2020 | Ta/Tis/T1 | **LNY <10:**  **5 years:** 60.6 (95% CI: 55.9−65.0) , **Log Rank** p<0.01  **LNY >=10:**  **5 years:** 68.7 (95% CI 66.0−71.1), **Log Rank** p<0.01 | - |
|  | Ta | **LNY <10:**  5 years: 69.0**, **Log Rank** p=0.82  **LNY >=10:**  5 years: 70.0**, **Log Rank** p=0.82 | - |
|  | Tis | **LNY <10:**  5 years: 62.5**, **Log Rank** p<0.12  **LNY >=10:**  5 years: 72.0**, **Log Rank** p<0.12 | - |
|  | T1 | **LNY <10:**  5 years: 60.0**, **Log Rank** p<0.01  **LNY >=10:**  5 years: 69.0**, **Log Rank** p<0.01 | - |
| Lyu 2024 | Ta/Tis/T1 | No significant difference between PLND and no PLND | - |
| Moldovan 2024 | Ta/Tis/T1 | **Overall Mortality Cox Regression (MVA):**  **PLND (vs no PLND):** 0.68 (0.62, 0.74), p<0.001 | - |
|  | Ta | **PLND**  5 years: 70.0**  **No PLND**  5 years: 60.0** | - |
|  | Tis | **PLND**  5 years: 70.0**  **No PLND**  5 years: 70.0** | - |
|  | T1 | **PLND**  5 years: 70.0**  **No PLND**  5 years: 53.0** | - |
| Von Landenberg 2018 | Ta/Tis/T1 | **Overall Mortality Cox Regression (MVA):**  **LNY >=10 (vs LNY <10):**  (No NAC)  0.49 (0.28–0.83), p=0.009 (NAC)  **LNY >=15 (vs LNY <15):**  0.89 (0.78–1.01), p=0.062 (No NAC)  0.69 (0.39–1.24), p=0.218 (NAC) | - |

** Extracted from Kaplan Meier Curves

**Supplementary Table 2:** Survival outcomes of clinical stage NMIBC
